# Rupture utérine sur utérus bicorne à 12 semaines d'aménorrhée: à propos d'un cas

**DOI:** 10.11604/pamj.2016.24.153.9697

**Published:** 2016-06-21

**Authors:** Sanaa Itchimouh, Karima Khabtou, Sakher Mahdaoui, Houssine Boufettal, Naima Samouh

**Affiliations:** 1 Service de Gynécologie Obstétrique ’'C’’, CHU Ibn Rochd, Maroc

**Keywords:** Utérus bicorne, rupture utérine, grossesse, Bicornuate uterus, uterine rupture, pregnancy

## Abstract

La fréquence des malformations utérines ayant un impact sur la reproduction est difficile à apprécier. Leur mise en évidence nécessite un bilan spécifique (hystérosalpingographie, hystéroscopie, cœlioscopie). La fertilité spontanée peut être altérée en fonction du type d'anomalie utérine. Toutes ces anomalies peuvent avoir des répercussions sur l’évolution de la conception à type de fausses couches précoces et tardives, de grossesse extra utérine, de menace d'accouchement prématuré, d'accouchement prématuré, de pathologies vasculaires gravidiques et de retard de croissance intra-utérin. L'utérus bicorne est la plus connue des malformations et représente environ la moitié des anomalies de l'utérus. La survenue d'une telle grossesse constitue une situation à risque pouvant entraîner une mort maternelle, mais le diagnostic précoce et un bon suivi peut mener des grossesses à terme sur des utérus malformé. Le dépistage échographique devrait permettre la détection systématique de ce genre de cas afin de prendre préventivement les mesures qui s'imposent. Nous rapportons un cas de rupture utérine sur utérus bicorne unicervical sur grossesse à 12 semaines d'aménorrhée.

## Abstract (en)

The incidence of uterine malformations affecting reproduction is difficult to assess. Their identification requires a specific assessment (hysterosalpingography, hysteroscopy, laparoscopy). Spontaneous fertility can be affected depending on the type of uterine abnormality. All these abnormalities can affect the evolution of pregnancy causing early and late miscarriage, ectopic pregnancy, threat of premature labour, premature labour, vascular pathologies during pregnancy and inadequate intra-uterine growth. Bicornuate uterus is the most common uterine malformation and represents about half of all uterine anomalies The occurrence of this type of pregnancy is associated with an increased risk of maternal mortality, but early diagnosis and proper monitoring can lead pregnancies to term on malformed uterus. Ultrasound screening should allow for a systematic identification of such cases in order to take the necessary preventive measures. We report a case of uterine rupture in a patient with unicervical bicornuate uterus at 12 weeks of amenorrhea.

## Introduction

Les malformations utérines sont relativement fréquentes puisqu'elles concernent 3-4% des femmes [1]. Heureusement, beaucoup d'entre elles sont asymptomatiques. Il est pourtant important d’évoquer ce diagnostic chez toutes patientes présentant une anamnèse de fausses couches à répétition, de fausses couches tardives ou d'accouchement prématuré, chez l'adolescente qui consulte pour une aménorrhée primaire, une dysménorrhée ou dyspareunie et chez les patientes suivies en médecine de la reproduction [2].

## Patient et observation

**Figure 1:**
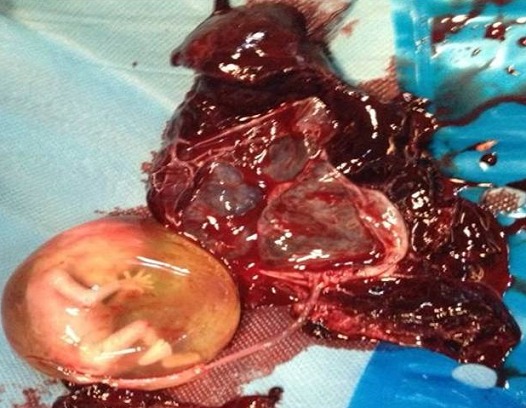
Fœtus d'environ 12 semaines d'aménorrhée avec placenta en intra-abdominal

**Figure 2:**
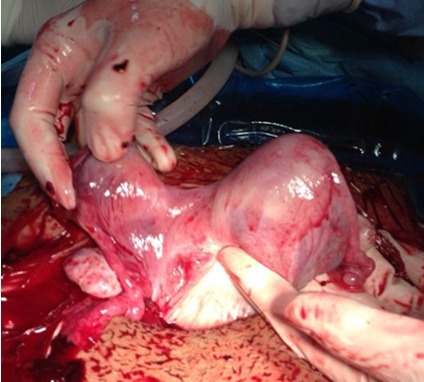
Vue per-opératoire d'un utérus bicorne

**Figure 3:**
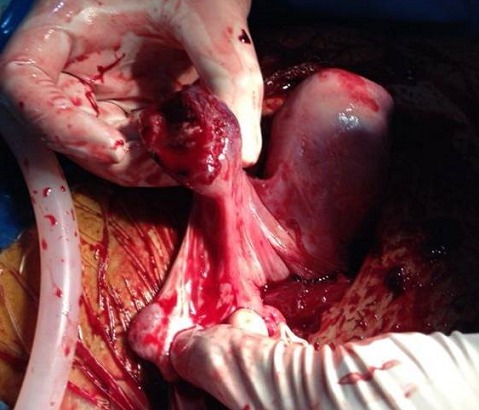
Aspect de l'hémi utérus droit rompu au niveau du fond

**Figure 4:**
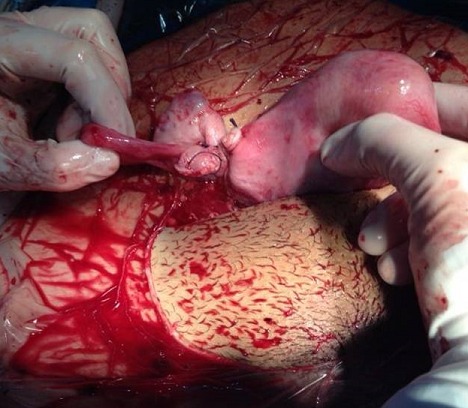
Réalisation d'une hémi-hystérectomie subtotale conservatrice

Il s' agit de Mme LR, âgée de 23 ans, 3^ème^ geste, 2^ème^ pare, ayant comme antécédents un enfant vivant par voie basse en présentation de siège et un décès néonatal (accouchement en présentation de siège à domicile avec rétention de la tête dernière), admise aux urgences pour prise en charge des algies pelviennes aiguës apparue depuis 24 h avant son admission sans métrorragies avec notion de retard de règles de 3 mois. L′examen à l′admission trouve une patiente consciente, apyrétique, les conjonctives légèrement décolorées, avec une tension artérielle à 10/6 et une fréquence cardiaque à 80 battements par minute. L′examen abdominal trouve une sensibilité pelvienne diffuse sans défense. L′examen gynécologique objective un seul col fermé, utérus légèrement augmenté de taille, pas de masse latéro-utérine. A l′échographie pelvienne: l′utérus augmenté de taille, présence en latéro-utérine droit d'un sac gestationnel contenant un embryon de 12 SA + 2 jours sans activité cardiaque, para ailleurs un épanchement de moyenne abondance est noté. Le taux de β-HCG (hormone gonadotrophique chorionique) plasmatique est revenu positif à 28602 UI/l. Le reste du bilan biologique révèle une anémie à 8,5 g/dl, avec taux de prothrombine à 59%. Le tableau évoque en premier le diagnostic de grossesse extra utérine. La patiente a bénéficié donc d′une minilaparotomie transversale: A l′exploration, présence d′un hémopéritoine de moyenne abondance de 700cc, avec fœtus d'environ 12 SA et placenta en intra-abdominal (Figure 1). Le reste de l′exploration révèle d'un utérus bicorne avec hémiuterus droit rompu au niveau du fond (Figure 2, Figure 3), d′où la décision de réaliser une hémi-hystérectomie subtotale conservatrice (Figure 4). Les suites post opératoires sont sans particularités et la patiente est déclarée sortante au 3^ème^ jour.

## Discussion

### Prévalence

L′incidence des malformations utérines congénitales dans la population féminine est estimée à 3-4%. Il est difficile de déterminer la prévalence exacte puisque beaucoup de ces malformations sont asymptomatiques et que les techniques d′imagerie telles que l′échographie 3D, l′hystérosonographie 3D et l′IRM ne sont disponibles que depuis quelques années. Les malformations utérines semblent être diagnostiquées plus fréquemment dans certains groupes de patientes, par exemple lors d′un suivi pour infertilité ou pour fausses couches à répétition [3]. L′utérus cloisonné est la malformation utérine la plus fréquente, comptant pour 30 à 50% des cas, suivie par les malformations utérines de type utérus bicorne et utérus unicorne.

### Organogenèse

Dès la 7 ^ème^ semaine du développement, les voies génitales féminines se différencient: en l′absence d′hormone anti-müllérienne, les canaux de Wolff régressent et les canaux de Müller vont se développer. Ce développement comporte trois phases: la migration des canaux de Müller vers le sinus urogénital (6^e^ à 9^e^semaine); l′accolement du tiers inférieur des canaux de Müller formant la cavité utérine et les deux tiers supérieurs du vagin (9^e^ à 13^e^semaine); la résorption de la cloison inter-müllérienne (13^e^ à 17^e^ semaine). La plupart des malformations utérines peuvent être expliquées par un défaut ou un arrêt du développement lors de ces trois phases: l′absence de migration ou la migration caudale incomplète des canaux de Müller vers le sinus urogénital sera responsable d′atrésies et/ou d′aplasies utérines complètes ou non; un défaut de fusion des canaux de Müller conduit à une duplication utérine (utérus didelphe, utérus bicorne); un défaut de résorption de la cloison intermüllérienne conduit à un utérus cloisonné. Un élément relativement constant est l′association d′anomalies de l′appareil génital et du système urinaire car l′embryogenèse de ces deux systèmes étant intimement liée.

### Grossesse et utérus bicorne

L′incidence des anomalies utérines congénitales dans une population fertile est de 3,2%, dont 90% sont des cloisons utérines et 5% soit utérus bicorne ou utérus didelphes [4]. L′utérus bicorne complique la grossesse, mais ne l′empêche pas. C′est souvent la grossesse elle-même qui révèle la malformation, car elle peut provoquer des avortements à répétition. Si les malformations utérines congénitales sont présentes chez 3-4% de la population féminine fertile et/ou infertile, leur fréquence s′élève à 5-10% chez les femmes consultant pour fausses couches à répétition et à 25% chez les femmes avec fausses couches tardives ou accouchement prématuré [5, 6]. Le problème chez ces patientes n′est pas celui de concevoir, mais de mener à terme la grossesse. Plusieurs facteurs expliquent cela: les malformations utérines sont associées à une cavité utérine de taille réduite, une musculature moins efficace, une incapacité de se distendre, une dysfonction myométriale et cervicale, une vascularisation inadéquate et un endomètre mal développé. Ces anomalies contribuent à un taux de fausses couches à répétition, d′accouchements prématurés, de présentations dystociques, de retard de croissance intra-utérin (RCIU) et de césariennes plus élevé; avec un risque accru de rupture utérine. [7].

### Prise en charge et traitement des utérus bicornes

La prise en charge des malformations utérines avant la grossesse comprend le traitement chirurgical pour autant qu′il soit indiqué et possible. Pour les utérus bicornes uni ou bicervicaux, la chirurgie réunificatrice des deux hémi-utérus, décrite par Strassmann en 1952, n′a pas montré de réel bénéfice [8]. Elle ne doit être réservée qu′aux patientes dont le pronostic obstétrical est extrêmement défavorable et dont l′anamnèse révèle plusieurs fausses couches tardives. Pour les utérus unicornes avec une corne rudimentaire controlatérale, le risque principal est de voir se développer une grossesse dans la corne rudimentaire, avec risque de rupture de l′hémi-utérus borgne. De ce fait, une résection de la corne rudimentaire est recommandée lorsqu′un endomètre est présent. Lorsque le diagnostic de malformation utérine est posé en début de grossesse, le traitement ne sera que préventif (repos, maturation pulmonaire, surveillance échographique de la croissance fœtale et de la compétence cervicale) [9]. Le cerclage cervical ne devrait être proposé qu′en cas d′incompétence cervicale prouvée, ce que l′on observe dans 25-30% des cas de malformations utérines [10].

## Conclusion

Les malformations utérines congénitales sont relativement fréquentes et souvent asymptomatiques. Chaque clinicien doit rechercher une malformation utéro-vaginale en présence d'une aménorrhée primaire, de douleurs abdominales, de fausses couches à répétition et dans certaines issues obstétricales défavorables. Il convient de rappeler que lors du diagnostic de malformation utérine, une imagerie des voies urinaires devrait être effectuée en raison des anomalies associées fréquentes. Un utérus bicorne ne conduit pas toujours à des complications mais il peut mener des grossesses à terme. Ce genre de malformation est très rare mais il importe d'en faire le diagnostic échographique de façon à gérer la situation préventivement et à permettre l'extraction des fœtus dans de bonnes conditions avant toute complication.
